# O080: Amplification of patient safety and infection prevention systems in southwest uganda: the power of district based in-hospital training

**DOI:** 10.1186/2047-2994-2-S1-O80

**Published:** 2013-06-20

**Authors:** I Spillman, M Ahimbisibwe, A Fry, SB Syed, S Hoyle, S Walker, T Tumwesigye

**Affiliations:** 1 Kisiizi Hospital, Church of Uganda, Kabale, Uganda; 2 African Partnerships for Patient Safety, World Health Organization, Geneva, Switzerland; 3 Countess of Chester Hospital, NHS, Chester, UK; 4 Uganda Protestant Medical Bureau, Kampala, Uganda

## Introduction

Kisiizi Hospital (KH) in partnership with Countess of Chester Hospital UK, as part of WHO African Partnerships for Patient Safety (APPS) has developed a programme to amplify crucial patient safety improvements to other district health facilities.

## Objectives

To describe an effective replicable mechanism of amplification of APPS.

## Methods

Using co-developed APPS resources, including in-house videos and demonstrations, training targeted all hospitals, training schools, and health centre IV’s within Rukungiri and Kabale districts. A Sensitisation Day aimed at key leaders of institutions was followed by a 2-day Training of Trainers seminar for those chosen to implement APPS principles. Patient safety, healthcare associated infections, hand hygiene, safe prescribing, WHO Safe Surgical Checklist, medical waste management, triage, monitoring, evaluation and teaching methods were covered, complimented by a tour of KH. Evaluations were completed and site-specific implementation plans formulated. Post-event site follow-up assessing progress with specific implementation goals is scheduled.

## Results

Training achieved 100% coverage for health centre IVs, hospitals, and district staff. In addition another regional hospital, two health educators, and four colleagues from Ndola Hospital, Zambia participated. Attendee evaluation was positive with 61% of attendees rating ten out of ten for overall quality of training. The tour and the varied methodologies utilised scored very highly.

## Conclusion

Key lessons emerge. First, success of regional coverage was due to good promotion and the innovative use of a “Sensitisation Day” to motivate leaders of institutions followed by appropriate staff selection to ensure implementation. Second, the Uganda-Zambia link enriched the training and proved mutually beneficial. Third, locating the seminars at an APPS hospital proved valuable in reality-focused training. Finally, comprehensive electronic resource compilation including in-house videos can provide support for on-going implementation. The process and amplification materials used can be replicated in other districts in Uganda and Africa to enhance patient safety.

## Disclosure of interest

None declared.

